# Evolution of Stress-Regulated Gene Expression in Duplicate Genes of *Arabidopsis thaliana*

**DOI:** 10.1371/journal.pgen.1000581

**Published:** 2009-07-31

**Authors:** Cheng Zou, Melissa D. Lehti-Shiu, Michael Thomashow, Shin-Han Shiu

**Affiliations:** 1 Department of Plant Biology, Michigan State University, East Lansing, Michigan, United States of America; 2 Department of Statistics and Probability, Michigan State University, East Lansing, Michigan, United States of America; 3 MSU-DOE Plant Research Lab, Michigan State University, East Lansing, Michigan, United States of America; The University of North Carolina at Chapel Hill, United States of America

## Abstract

Due to the selection pressure imposed by highly variable environmental conditions, stress sensing and regulatory response mechanisms in plants are expected to evolve rapidly. One potential source of innovation in plant stress response mechanisms is gene duplication. In this study, we examined the evolution of stress-regulated gene expression among duplicated genes in the model plant *Arabidopsis thaliana*. Key to this analysis was reconstructing the putative ancestral stress regulation pattern. By comparing the expression patterns of duplicated genes with the patterns of their ancestors, duplicated genes likely lost and gained stress responses at a rapid rate initially, but the rate is close to zero when the synonymous substitution rate (a proxy for time) is >∼0.8. When considering duplicated gene pairs, we found that partitioning of putative ancestral stress responses occurred more frequently compared to cases of parallel retention and loss. Furthermore, the pattern of stress response partitioning was extremely asymmetric. An analysis of putative *cis*-acting DNA regulatory elements in the promoters of the duplicated stress-regulated genes indicated that the asymmetric partitioning of ancestral stress responses are likely due, at least in part, to differential loss of DNA regulatory elements; the duplicated genes losing most of their stress responses were those that had lost more of the putative *cis*-acting elements. Finally, duplicate genes that lost most or all of the ancestral responses are more likely to have gained responses to other stresses. Therefore, the retention of duplicates that inherit few or no functions seems to be coupled to neofunctionalization. Taken together, our findings provide new insight into the patterns of evolutionary changes in gene stress responses after duplication and lay the foundation for testing the adaptive significance of stress regulatory changes under highly variable biotic and abiotic environments.

## Author Summary

Plants have developed a multitude of response mechanisms to survive stressful environments. Since the environment is highly variable, these stress response mechanisms are expected to undergo frequent innovation. Duplicate genes represent a potential source for such innovation. In this paper, we explored the evolutionary changes in stress responses at the transcriptional level among duplicated genes in the model plant *Arabidopsis thaliana*. We found that after gene duplication, ancestral stress responses tend to be retained by only one of the gene duplicates (partitioning). In addition, the pattern of partitioning of multiple stress responses is extremely asymmetric, where one duplicate tends to inherit most or all of the ancestral stress responses. We present evidence that the asymmetric loss of stress responses is correlated with the asymmetric loss of putative transcription factor binding sites. Interestingly, those duplicate genes inheriting few or no ancestral responses tend to have gained new stress responses, providing support for the model that gene duplicates are a source of innovation. Our findings provide important insight into the mechanisms of gene function evolution and lay the foundation for experimental studies to determine the significance of gain of stress responses in plant adaptation.

## Introduction

The ability to sense and respond properly to environmental stresses, such as cold, draught, wounding and biotic interactions, is central to the survival of all living organisms. The selection pressures imposed by these stresses and sedate nature of plant life histories have likely led to the evolution of elaborate mechanisms in plants to cope with stresses [1]–[4]. Given that environmental conditions are highly variable, stress sensing and response mechanisms are expected to change rapidly and require constant innovation. One potential source of such innovation is from duplicate genes, which have been hypothesized to be a main source of evolutionary novelties [5]. Given that gene and genome duplications likely have contributed significantly to the morphological complexity in plants [6], they may influence physiological complexity in stress responses as well. Consistent with this view, the gene duplication rate appears to be substantially higher in plants compared to most other organisms [7]. A large number of plant duplicates have been retained for tens of millions of years [8]–[13]. In addition, such duplicated genes tend to be over-represented in stress-related functional categories [14],[15]. Analyses of a stress expression dataset from *Arabidopsis thaliana* revealed that duplicate genes derived from lineage-specific expansion events tend to be involved in responses to environmental stimuli [13]. These findings indicate that plant gene duplication and retention are strongly biased toward genes involved in stress response. This relationship highlights the importance of examining the patterns of stress response changes after gene duplication to better understand the evolution of stress responses in plants.

We previously showed that plant stress responsive genes tend to have a higher rate of retention after duplication than non-responsive genes [13], but it is not clear which mechanisms contribute to their retention. After duplication, the predominant fate of duplicates is pseudogenization [5],[16]; however, a significant fraction of gene duplicates are preserved. Several alternative but not mutually exclusive models have been proposed to explain their preservation. The classic neofunctionalization model proposed by Ohno asserts that, after duplication, one duplicate may retain the ancestral functions while the other occasionally attains a new role [5]. The neofunctionalization model predicts that the two duplicate copies evolve in an asymmetric fashion with one duplicate experiencing faster and positive evolution with potentially novel functions [11],[17],[18]. In addition to neofunctionalization, processes that do not involve positive selection may also lead to retention of duplicate genes [19],[20] and rate asymmetry [21]. One intriguing alternative explanation for non-adaptive retention of duplicates is based on the duplication-degeneration-complementation (DDC) model [20],[22] and a similar model proposed by Stoltzfus [23]. The DDC model postulates that after duplication, both duplicates may have complimentary ancestral functions (subfunctionalization) retained through negative selection. There are quite a few studies demonstrating partitioning of gene functions consistent with the DDC model [11], [22], [24]–[30]. Inconsistent with DDC, however, evolutionary rates of duplicate coding sequences tend to be highly asymmetric [18],[31]. In addition, studies on divergence of protein-protein interaction partners [32]–[34] and expression divergence [32],[35],[36] between duplicate genes have shown that sub-functions tend to be distributed among duplicates in an extremely asymmetric fashion. Therefore, many gene pairs do not appear to have complementary functions as expected under the DDC model. As a result, it has been suggested that duplicate retention can be explained by both the neofunctionalization and DDC models and that subfunctionalization is typically followed by neofunctionalization [21],[37],[38].

Although studies on the putative mechanisms of duplicate retention have generated a number of insights, one major issue is that they do not explicitly consider the ancestral functional states. For example, in earlier studies, an ancestral gene was assumed to have the same spatial-temporal or condition-specific expression pattern as the extant gene. This is overly simplistic since the extant gene function can be a consequence of functional gain. In addition, defining whether genes experienced neofunctionalization and subfunctionalization (based on the DDC model, [22]) require comparisons between the functional states of extant genes against those of their ancestors. Ancestral character reconstruction has been an important focus in molecular evolutionary study, yet few studies have estimated ancestral states to examine expression divergence [39],[40] and, more specifically, the gain and loss of expression patterns [41] among duplicate genes.

In this study, we investigated stress response evolution after duplication to further our understanding of the evolutionary trajectories of ancestral gene functions. Using *Arabidopsis thaliana* stress expression data, we set out to explore how gene duplication impacts the stress response evolution of *A. thaliana* paralogs. We estimated ancestral stress response states, which is crucial for determining the nature of evolutionary changes. We found substantial changes in stress response including losses and gains among paralogous genes. In addition, the putative ancestral expression patterns were partitioned among duplicate gene pairs in a highly asymmetric fashion. This asymmetry in expression can be, at least in part, attributed to asymmetry in *cis*-regulatory elements that are likely involved in stress responses. Finally we found that sub-functionalized duplicate pairs tend to undergo neofunctionalization, providing additional support for the hypothesis that subfunctionalization and neofunctionalization are both required to explain retention for at least some of the duplicated pairs.

## Results

### Integration of expression and phylogenetic data for inferring putative ancestral expression states

**Figure 1:**
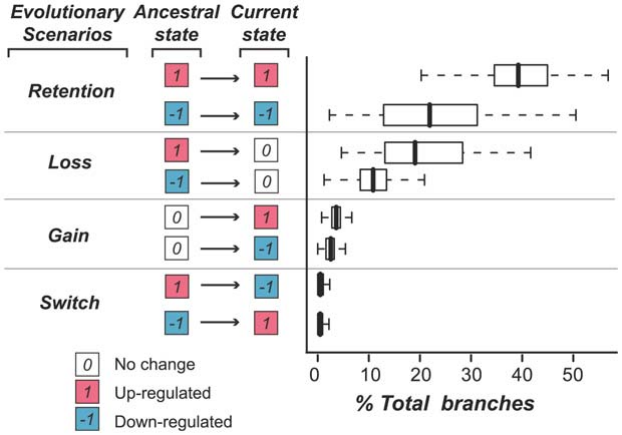
Preponderance of stress response evolution scenarios. We considered four possible stress response evolution scenarios of ancestral-extant gene pairs including retention, loss, gain, and switch. The gene pairs were defined based on external branches of gene family phylogenies. The “ancestral” column shows the inferred stress responses of the ancestral genes, and the “extant” column indicates the stress responses of extant genes. The distribution of percentages of external branches exhibiting a particular scenario over all conditions is plotted as a box plot on the right. 1: up-regulated under stress (red), −1: down-regulated under stress (blue), 0: non-responsive.

The elevated rate of duplication and retention of stress responsive genes suggests that duplicate genes are a source of crucial innovations important for proper responses to stressful environments [13]. After duplication, one or both duplicates may retain or lose their original functions or gain new functions (Figure 1). What is the relative abundance of these distinct evolutionary scenarios? Most importantly, how frequently has innovation occurred in the context of stress response? To address these questions, we integrated stress expression data and information on the phylogenetic relationships between paralogs of *A. thaliana* to evaluate stress functional evolution of duplicate genes. Specifically, we focused on AtGenExpress gene expression data collected using samples grown under 16 abiotic and biotic stress conditions (see Methods). For each condition, genes that are responsive (significant up or down-regulation relative to the controls) were identified. These response states were then mapped to gene family phylogenies generated using a Maximum Likelihood (ML) method [42]. Ancestral stress responses were estimated according to the stress response patterns of and the phylogenetic relationships between duplicates for every *A. thaliana* gene family.

Multiple methods for character state reconstruction have been developed including Maximum Parsimony (MP) [43], ML [44],[45], and Bayesian inference (BI) [46]–[48]. There are two major issues in ancestral state inference [49],[50]. The first is the uncertainty in character mapping due to multiple, equally likely ancestral states (mapping uncertainty). The other issue in character state reconstruction is phylogenetic uncertainty: the uncertainty in the relationships between genes due to potential errors in phylogenetic reconstruction. Mapping uncertainty is the major disadvantage in using MP while phylogenetic uncertainty is a major issue for MP and ML. BI is the only method that addresses the phylogenetic uncertainty problem [46],[49]. In this study, we used the ML method to construct ancestral states using gene family phylogenies generated via a Bayesian method (see Methods) instead of BI for the following reasons. The first is that it is difficult to use the BI method for genome-wide studies due to significant computational costs. The second reason is that we used a Bayesian method to infer gene family phylogenies, which accounts for the phylogenetic uncertainty in a similar fashion as the BI approach. Thirdly, the ML method, similar to BI, provides an assessment of the reconstruction accuracy in a probabilistic format and has been shown to provide more accurate estimates than MP [51]. Finally, ML has been widely used in character state reconstructions in molecular evolutionary, ecological, and systematics studies [52],[53].

We should emphasize that, like phylogenetic reconstruction, ancestral state inferences should be treated as hypotheses of potential ancestral conditions. In addition, two major assumptions were made that may impact the reconstruction outcomes. First, we assumed that evolutionary distance (tree branch length) is positively correlated with the probability of character state change. To test how this assumption may impact our findings, the ancestral states were also estimated for all gene families without this correlation assumption. We found that the likelihood values generated with and without the correlation assumption are not significantly different (Figure S1), suggesting that this assumption is unlikely to affect our result significantly. Another assumption is rate constancy, that is, the rate of changes are the same among different branches of the tree [54],[55]. Currently, no character state reconstruction method allows variable rates of evolution, an issue that is currently being addressed [Mark Pagel, personal communication]. Nevertheless, our study represents an initial attempt to explore stress response evolution based on ancestral states reconstruction. As is apparent in later sections, findings that rely on the predicted ancestral states are consistent with multiple hypotheses in duplicated gene evolution. Moreover, several of our major conclusions are supported with or without the estimation of ancestral states.

### Comparisons of stress responsiveness between extant and ancestral genes

In this study, we only considered stress response status of ancestral nodes immediately leading to extant genes in all subsequent analyses. This is because the ancestral stress responses of nodes between internal branches must be estimated based on the stress response of another prediction and not directly from extant genes, which may introduce more error into the analysis. Comparisons are also difficult among internal nodes that link branches with variable numbers of duplication events, even if the duplicate events in question took place at approximately the same time.

There are 14,001 genes significantly up and/or down-regulated (with 5% false discovery rate) under at least one stress condition. Of these, 11,203 (∼80%) are gene family members. In this analysis we looked at the stress response for each stress condition and time point independently. Therefore, a “stress evolution event” is defined according to a comparison between the stress response of an extant gene and the putative response of its closest ancestral node in a gene tree for each stress condition/time. We found that ∼10% (37,413 out of 356,556) of all possible evolution events satisfy at least one of the following criteria: (1) extant genes are responsive to ≥1 stress conditions and (2) ancestral genes are predicted to be responsive to ≥1 stress condition. Among these stress related events, 61% (median across all conditions/time points) of extant genes have retained the same stress responsiveness as their parental genes (either up-regulated, 1→1 (39%), or down-regulated, −1→−1 (22%), Figure 1). In contrast, only 30% of extant genes experienced loss of responsiveness (1→0: 19% or −1→0: 11%). If loss of function occurred quickly after gene duplication, we would expect ∼50% of duplicates would have lost their ancestral stress response. Stress response gain occurred in 6% of the external branches (0→−1 or 0→1, Figure 1), indicating that gain of function in the form of up- or down-regulation under stress conditions, although less frequent than loss of responses, occurs readily. These putative stress response gains may serve as the source of evolutionary innovations required for adaptive evolution under stress conditions. Finally, only in relatively few cases (2%) did stress response “switch” occur (1→−1 or −1→1, Figure 1), suggesting that a stress response switch likely involved a loss event followed by a gain in response.

We found that the relative abundance of these four evolutionary scenarios is similar among stress conditions (Table S1); therefore, only the distributions across all conditions are shown in Figure 1. Our findings highlight the importance of ancestral state reconstruction in studying functional evolution of duplicate genes. Without considering putative ancestral states, these stress responsiveness loss and gain events would be indistinguishable. Taken together, we found that stress response evolution in *A. thaliana* is highly versatile with a large number of duplicate genes experiencing gains or losses of stress responsiveness.

### Temporal patterns of stress response evolution

**Figure 2:**
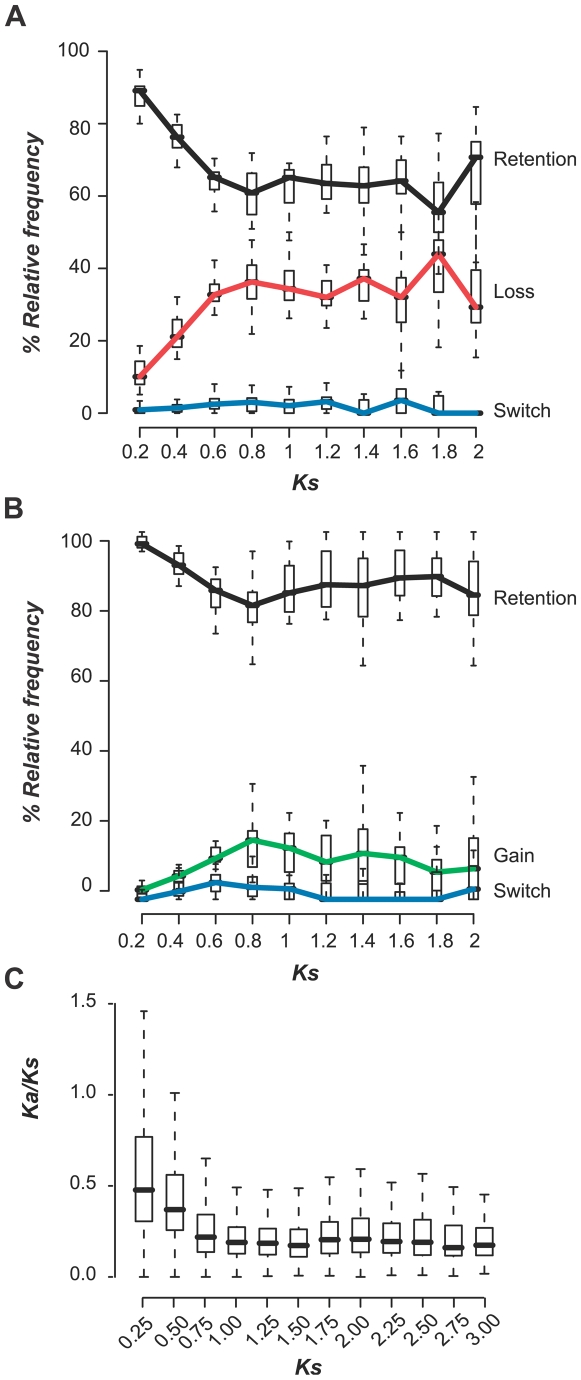
Relationship between stress evolution scenarios and Ks. (A) The relative abundance of external branches exhibiting retention, loss, and switch of stress response was determined for each of the 8 abiotic stress conditions. Here we only show ancestral-extant pairs where the ancestral gene was up-regulated in ≥1 conditions. In each Ks bin, the relative frequency of each stress evolution scenario (number of extant genes with retention, loss, or switch over the total number of extant genes) is summarized as a box plot (including values from all conditions/times). A colored line connects the median values to indicate the trend over Ks. Black line: retention. Red: loss. Switch: blue. (B) The relative abundance of external branches with retention, gain, and switch under abiotic stress conditions. Only ancestral-extant gene pairs where extant genes were up-regulated in ≥1 conditions were considered. Boxplots were generated as described in (A). Black line: retention. Green: gain. Blue: switch. Similar plots for biotic stress conditions, for down-regulated genes, and for each condition are shown in Figure S2. (C) Ka/Ks-over-Ks plot of *A. thaliana* duplicates (reciprocal best match within species). The Ka/Ks values were separated into multiple Ks bins. For all box plots: black bars indicate median values, boxes delineate 1st and 3rd quartiles, and dotted lines delineate 1–99 percentiles.

In the previous section we showed that ∼63% of the extant genes retained their ancestral stress responses (Figure 1). One possible explanation for this high rate of retention is that the duplicate genes we analyzed tend to be derived from more recent duplication events. Given that there is a positive correlation between the degree of expression divergence of duplicated genes and their divergence times [56], the degree of stress response change is likely correlated with the age of gene duplication events as well. To determine if younger duplicates are more likely to have retained stress responsiveness, we analyzed the relative frequencies of the different stress response evolution scenarios (as shown in Figure 1) among gene duplicates using synonymous substitution rate (*Ks*) as a proxy for time. Since the findings are in general true regardless of whether we examine abiotic or biotic stress data, whether we focus on up- or down-regulation, or whether we examine each condition independently (see Figure S2), we summarized the relative abundance of branches over all abiotic or all biotic conditions as boxplots for each evolutionary scenario and *Ks* bin. As an example, the results for up-regulation under abiotic stress conditions are shown in Figure 2. We found that, regardless of *Ks* (at least when *Ks*<2), the relative abundance is: retention>loss≫switch (Figure 2A). However, the relative abundance is not constant over time. Soon after the duplication, the overall proportion of response loss increases rapidly when *Ks*<0.8 (Figure 2A). Most interestingly, after *Ks* reaches approximately 0.8 (Figure 2A), the proportion of duplicates with response retention and loss becomes relatively stable.

Younger duplicates tend to experience accelerated evolution at the coding sequence level, presumably due to relaxation of purifying selection [57],[58]. The *Ka/Ks* decline observed in a *Ka/Ks*-over-*Ks* plot of *A. thaliana* duplicates is similar to the pattern of response loss (Figure 2C). This pattern of stress response evolution over time can be due to the fact that we modeled ancestral state evolution after that of coding sequences. However, similar results were also obtained when we inferred ancestral states without assuming a correlation between sequence and functional state evolution (data not shown).

The predominant fate of duplicate genes is loss, and the half-life of duplicates is several million years [59]. If an ancestral “sub-function” (responsiveness under a particular stress condition) found in a pair of duplicate genes is completely identical, we may expect that the sub-function in question will eventually be lost in one of the duplicate copies (∼50% of duplicates). Intriguingly, we found that ∼60% of duplicate lineages retain stress responsiveness even when *Ks* is as large as 2.0. Based on the silent substitution rate estimate of ∼0.6e^−3^/site/million years, [60], our finding suggests that a substantial number of duplicate pairs maintain the same stress responses over hundreds of millions of years. One interpretation is that “redundant” stress response(s) are maintained in duplicate pairs over a very long period of time. This is consistent with the “functional buffering” hypothesis stipulating that genes with crucial functions tend to be retained to ensure the essential functions can be carried out in the event of inactivation of one duplicate [61]. However, this explanation appears to contradict the finding that “essential” genes tend to be singleton genes [62]. Another possibility is that the stress responses of a duplicate pair are qualitatively different even though both duplicates are classified as responsive. Further analysis would be necessary to distinguish between these possibilities.

We next conducted the same analysis on branches with an up-regulated extant gene to examine the dynamics of response gain over time (Figure 2B). Similar to the patterns observed when looking at stress response loss/retention over time (Figure 2A), there is an initial increase in the proportion of lineages with stress response gains after duplication, but this proportion remains relatively steady when *Ks*>0.8 (Figure 2B). It is known that the “functional similarity” between duplicate genes decreases as *Ks* increases. For example, in multiple eukaryotic species, the expression correlation between a duplicate pair decreases as *Ks* increases [56], [63]–[65]. Similarly, the fraction of potential protein interaction partners (predicted based on co-expression) shared between a duplicate gene pair in human declines as *Ks* increases [34]. Therefore, it is not particularly surprising that the overall proportion of duplicates that lose stress responsiveness increases as *Ks* increases. The intriguing aspect is that, similar to the loss of stress response, the rate of response gain is much higher at early periods after gene duplication but slows down later on. As with loss of stress responsiveness, this initially high gain rate is likely the consequence of relaxation of selection after duplication. The probability of a duplicate gene gaining novel stress responses diminishes over time, potentially due to increasingly strong purifying selection on the retained duplicates (Figure 2C).

### Stress response evolution scenarios and mechanisms of duplication

**Figure 3:**
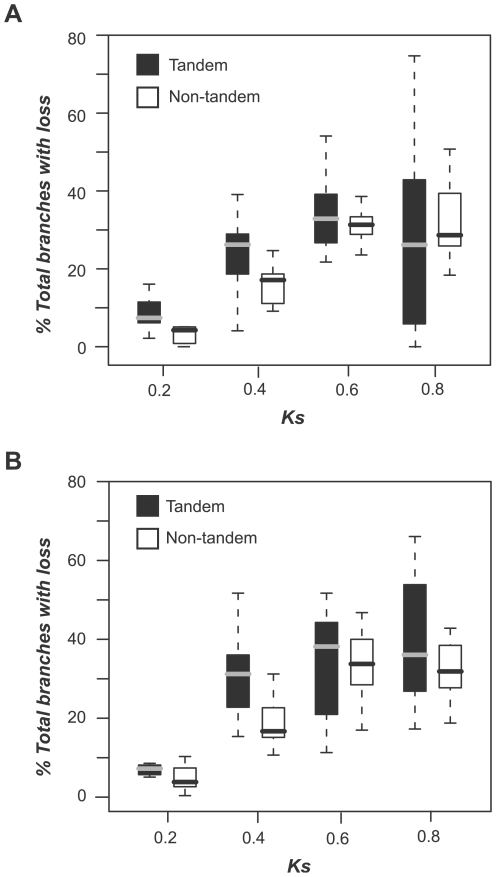
Extent of stress response loss among duplicates derived from different duplication mechanisms. (A) Comparison of the extent of loss of up-regulation under abiotic stress conditions for genes derived from tandem and non-tandem mechanisms. For each Ks bin and duplication mechanism, a box plot was generated using the percentage of total ancestral-extant gene pairs (external branches) with response loss (loss of up-regulation) among all abiotic conditions. Black: tandem duplicates. White: non-tandem duplicates. Asterisks indicate significant differences between percent loss distributions of tandem and non-tandem duplicates based on Wilcoxon rank sum test. (B) Comparison of the extent of loss of up-regulation under biotic stress conditions for genes derived from tandem and non-tandem mechanisms. Similar plots for down-regulation of duplicates are shown in Figure S2. Asterisks indicate significant differences based on Wilcoxon rank sum tests (p<0.05).

Stress responsive genes tend to be located in tandem clusters [12],[13]. Therefore, in addition to the timing of gene duplication, it is expected that another important factor in the evolution of stress responsiveness may be duplication mechanism, or whether duplicates are derived from tandem or non-tandem duplication mechanisms. Tandem duplication occurs via unequal crossing over during recombination, and there is variability in the size of the intergenic sequences that are duplicated. As a result, dramatic changes in gene expression patterns between duplicates might be expected. In contrast, the entire gene “neighborhood” is duplicated during whole genome duplication (WGD), which has given rise to the majority of non-tandem duplicated genes. Therefore, the expression patterns of WGD duplicates may be more similar. Consistent with these expectations, we found that the percentage of tandem duplicated genes experiencing loss of up-regulation under stress is greater than that of non-tandem duplicates when *Ks*<0.6 (for abiotic and biotic conditions, Figure 3A and 3B, respectively). Similarly, the proportion of tandem duplicates experiencing loss of down-regulation under stress conditions is also consistently larger than that of WGD duplicates (Figure S3). It should be noted that the difference in the percentage of tandem and non-tandem duplicates experiencing loss and gain is significant when *Ks* = 0.4, which coincides with the most recent WGD event in the *A. thaliana* lineage [12],[66], regardless of the conditions (abiotic and biotic) or the mode of regulation (up or down-regulated under stress; Figure 3; Figure S3). In addition to stress response loss, we examined gain of response among duplicates derived from tandem and non-tandem mechanisms. Similarly, proportionally more tandem duplicates tend to experience stress response gain. However, because there are relatively few cases of gains compared to losses, we do not have strong statistical support for most *Ks* bins.

Our results illustrate that duplication mechanism affects the pace of stress response evolution, particularly the pattern of response loss. Specifically, tandem duplicated genes likely experience more frequent stress response loss than non-tandem duplicates. This is consistent with our assumption that tandem duplicated genes are subject to more dramatic changes in their sequence content when duplicated. Our finding is also consistent with an earlier observation that the expression correlations between *A. thaliana* duplicates created by “large-scale”, presumably whole-genome, duplication events are stronger compared to those between duplicates derived from “small-scale”, predominantly tandem, duplications [35].

### Substantial stress response partitioning among duplicated pairs

**Figure 4:**
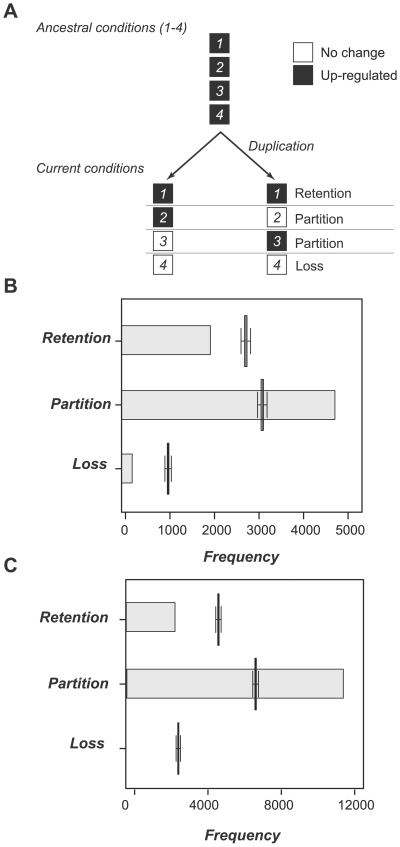
Stress response evolution of duplicate pairs. (A) An ancestor-duplicate gene pair and the three possible scenarios (retention, partition, and loss) of stress response evolution. Note that the stress conditions (1–4) considered here are all up-regulated (black boxes) in the ancestral gene. Results for down-regulated conditions are shown in Figure S3. Here “retention” and “loss” are defined as situations where both duplicates retain or lose a particular stress response. (B) The observed frequency of each scenario shown in (A) when ancestral states were considered. The bar plots indicate the observed frequency and the box plots indicate the frequency distributions of random scenarios. The random scenarios were generated by assigning the extant genes to stress responses randomly and determining the frequency of each stress response scenario over 10,000 runs. (C) The observed frequency of each scenario shown in (A) without considering ancestral states. The box plots were generated based on the same randomization scheme as in (B).

Based on analyses of the stress response evolution of ancestral and extant genes, we found that the relative proportion of duplicates that retain stress responsiveness vs. those that lose stress responsiveness is ∼60% after *Ks* reaches 0.8 (Figure 2). This implies that both duplicates in some duplicate pairs have retained ancestral stress responsiveness (parallel retention) while in other duplicate pairs loss of responsiveness occurred in both (parallel loss) or just one duplicate (partition) (Figure 4A). To directly evaluate the frequencies of these different scenarios, we examined the evolution of stress response changes among duplicate pairs. Analysis of gain-of-function among duplicate pairs is discussed in later sections. Response switch was not considered because it occurs much less frequently than other possible stress response changes.

Here parallel retention (referred to as retention) of a stress response refers to a situation where both of the daughter duplicate genes maintain the same stress response as their ancestor. Partitioning indicates that only one of the duplicates has the same stress response as the ancestral gene. Parallel loss (referred to as loss) describes the scenario where none of the progenitors retain the ancestral stress response. The frequencies of each evolutionary scenario among duplicate pairs with ≥1 informative conditions (conditions where ancestral genes are predicted to be up-regulated) are shown in Figure 4B. Note that an ancestral gene and its daughter duplicates may be classified into ≥1 scenarios because the ancestor was responsive to ≥1 stress conditions (Figure 4A). We found that there are significantly more duplicate pairs experiencing partitioning than the random expectation (*p*<1E-06). On the other hand, the numbers of duplicate pairs experiencing both parallel retention (*p*<1E-06) and loss (*p*<1E-06) are significantly less than expected randomly. Similar trends in down-regulation were found as well (Figure S4). We have also performed a similar analysis without considering ancestral states and found that partition is over-represented and retention is under-represented (Figure 4C). Therefore, with or without ancestor reconstruction, our results indicate that stress response partitioning is the predominant fate of duplicated gene pairs.

### Patterns of stress response partitioning among duplicates

**Figure 5:**
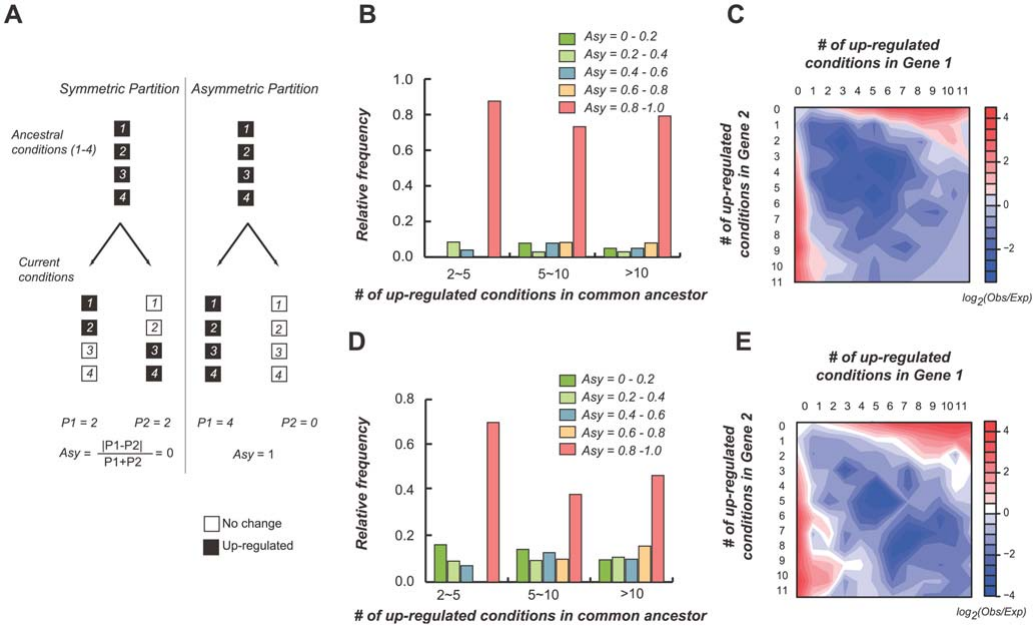
Extreme asymmetry in ancestral stress response partitioning among duplicates. (A) Asymmetry measure (*Asy*) definition. *Asy* was calculated based on the number of conditions, P, that each duplicate is responsive to. P1 is defined as the maximum number of responsive conditions among the two duplicates pairs. P2 is the minimum number. Extreme asymmetry was defined based on *Asy* = 1. (B) Relative frequencies of the number of duplicate pairs with various degrees of asymmetric partitioning. Here we only considered ancestrally up-regulated conditions. A similar plot for down-regulated conditions is shown in Figure S4. To demonstrate how *Asy* was affected by the number of conditions examined, the analyses were conducted using three datasets, each with a different number of informative conditions. (C) Over-representation of duplicate pairs with extremely asymmetric partitioning of stress responses with ancestral states taken into consideration. Log ratio (base 2) between the observed and expected number of duplicate pairs for each condition combinations was used as a measure of over- (red) and under-representation (blue) in the contour plot. The expected numbers were generated based on random binomial sampling. (D) Calculation of the relative frequencies of the number of duplicate pairs with various degrees of asymmetric partitioning without inferring ancestral states (up-regulation of extant genes is assumed to be ancestral). (E) Over-representation of duplicate pairs with extremely asymmetric partitioning of stress responses without considering ancestral states.

Why are there significantly more cases of stress response partitioning between duplicate pairs than retention and parallel loss? One explanation is that duplication released one copy from purifying selection and random mutations accumulated that eventually lead to loss of function [5]. Another explanation is that subfunctionalization has occurred as defined in the DDC model [20]. The DDC model stipulates that duplicate gene retention is due to subfunctionalization where each of the genes in a duplicate pair specializes in a subset of the ancestral functions [20]. If we consider response under each condition as a “subfunction”, the substantial partitioning of ancestral stress responses suggests frequent subfunctionalization (Figure 4B). To assess the frequency of duplicate pairs with subfunction partitioning patterns consistent with the predictions of the DDC model, we analyzed stress response partitioning patterns of each duplicate pair and its ancestral gene by examining all informative conditions as shown in Figure 5A. Here an informative condition is defined as a stress condition where (1) the ancestral gene is predicted to be responsive and (2) this ancestral response is partitioned among the daughter duplicates.

Subfunctionalization of the duplicated pairs requires that the duplicates maintain ≥1 subfunctions of their ancestors and that partitioning is to some degree symmetric. To measure the degree of asymmetry of subfunction partitioning, we have devised a measure, *Asy* (Figure 5A). *Asy* for a duplicate pair is 1 if ancestral responses of all informative conditions are partitioned into only one of the duplicates (asymmetric partitioning, Figure 5A). *Asy* is 0 if the partitioning of ancestral subfunctions occurs equally among two genes. Symmetric partitioning consistent with the DDC model is defined as *Asy*<1. Interestingly, we found that in most duplicate pairs stress response partitioning is extremely asymmetric no matter how many informative conditions were examined (up-regulated ancestral conditions, Figure 5B; for down-regulated responses, see Figure S4). To test for over-representation of duplicate pairs exhibiting different types of asymmetry, we looked at the log ratios between the observed and randomly expected numbers of duplicate pairs exhibiting different degrees of asymmetry (Figure 5C). Consistent with the interpretation that subfunction partitioning is extremely asymmetric, we found enrichment of gene pairs in all combinations where gene 1 in a pair is responsive to ≥2 stress conditions but gene 2 in the same pair is responsive to none or very few conditions. Similar results were obtained without considering ancestral states (Figure 5D and 5E). This pattern of extreme asymmetry is also true among down-regulated genes (Figure S5). Our findings indicate that, although in some duplicate pairs the stress response partitioning pattern is consistent with the prediction of DDC model, in most cases the partitioning is extremely asymmetric (with *Asy* = 1).

Although our observations may contradict the prediction of the DDC model that there should be little or no extreme asymmetry in subfunction partitioning, they are consistent with earlier studies in which asymmetric divergence in *Ka/Ks* ratios, gene expression patterns and co-regulation networks were observed [32], [34]–[36],[40]. One of the major differences between our study and earlier studies is that we consider putative ancestral states. Nonetheless, we still reach a similar conclusion as earlier analyses. This can be attributed to our finding that gain of stress response does occur much less frequently compared to response loss (Figure 1). One limitation of our (or any) analysis regarding subfunction partitioning is that only a subset of the potential subfunctions has been examined. Note that when the number of informative stress conditions increases (Figure 5A), the number of duplicates with evidence of subfunctionalization increases as well (*Asy*<1, Figure 5B). Therefore, we cannot rule out the possibility that some duplicate pairs with extreme asymmetry in fact have complementary subfunctions that are yet to be discovered.

In addition to the trivial explanation that asymmetry is due to insufficient number of conditions examined, a neutral model has been proposed to explain the preponderance of functional asymmetry among yeast duplicates [32]. In this model, asymmetric divergence is expected to be more prominent in species that have larger effective population sizes such as yeast [32]. However, human duplicates diverge rapidly in their potential protein interaction partners in a highly asymmetric fashion even though the effective population size is much smaller than that of yeast [34]. In *A. thaliana*, which is a selfing species with an effective population size of 1, expression divergence of duplicates is highly asymmetric ([36] and this study). Therefore, it appears that the neutral model suggested by Wagner [32] may not be the whole story and adaptive evolution in the form of neofunctionalization may contribute to stress response asymmetry.

### Asymmetric partitioning pattern of stress responses and putative *cis*-elements

**Figure 6:**
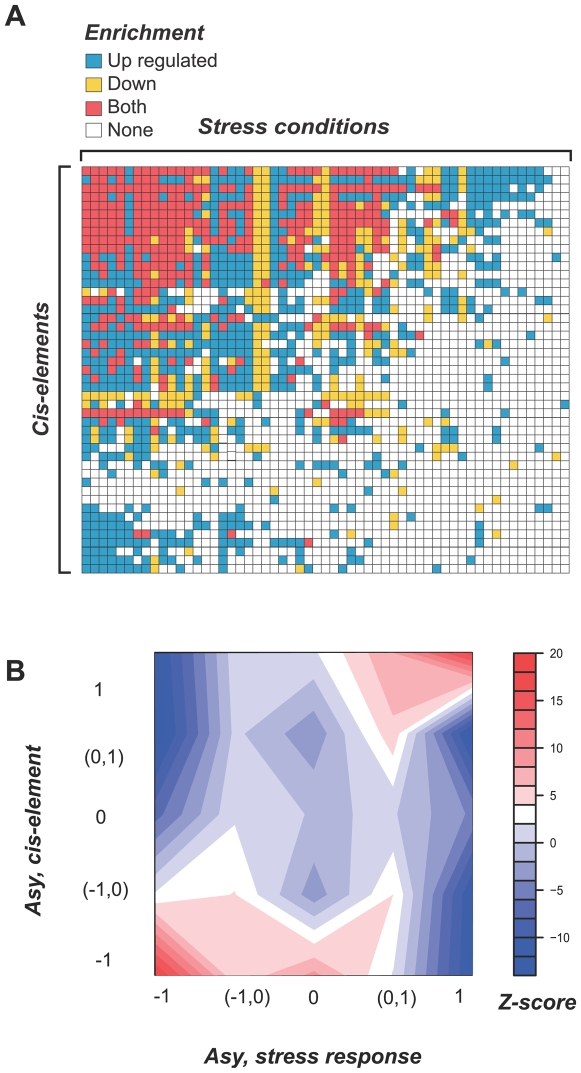
Correlation between stress response and *cis*-regulatory evolution. (A) Enrichment of putative *cis*-elements in the putative promoter regions of stress responsive genes under different conditions (includes different time points for each treatment, e.g., drought 3 hr, drought 6 hr, etc.). Significant enrichment of a particular *cis*-element in the promoters of up-regulated, down-regulated, or both up- and down-regulated genes is indicated by blue, yellow, and red, respectively. No significant enrichment under a condition is indicated by a white box. (B) Contour plot illustrating the positive correlation between stress response and putative *cis*-element asymmetries. *Cis*-element *Asy* is defined in the same way as for stress response partitioning (Figure 5A) except that, presence (1) or absence (0) of a putative *cis*-element was used in place of up-regulation (1) and no change (0). For a particular duplicate pair, we randomly assigned index 1 and 2 to the duplicates, so that when we calculated asymmetry score (*Asy*, Figure 5A) for partitioning of responsiveness and *cis*-elements, the subtractions were in the same direction. As a result, half of the time *Asy* is below zero. The observed number in each *Asy* value bin was compared to simulated datasets generated by random assignment of putative *cis*-elements among extant genes while fixing the number of genes with a particular element. The deviation of the observed numbers from random expectation was assessed by calculating the Z-score. Shades of red and blue indicate over and under-representation, respectively.

Our results show that asymmetric partitioning of stress responses is a predominant feature of plant stress response evolution. However, the molecular basis for asymmetric partitioning is unknown. Several studies have shown a positive correlation between expression divergence and *cis*-regulatory motif divergence [67],[68]. In addition, mutations in *cis*-regulatory regions have been hypothesized to serve as the mechanistic basis for subfunctionalization [20],[69]. Therefore, we set out to determine if stress response partitioning is correlated with *cis*-regulatory element content in the promoter regions of duplicate genes. Specifically, we asked if duplicate pairs with asymmetric partitioning of stress responses also tend to have asymmetric partitioning of *cis*-elements. To identify *cis*-elements that are potentially important for controlling expression under the stress conditions we examined, we first mapped known plant *cis*-elements to the putative promoter regions of *A. thaliana* genes (see Methods). Note that we did not verify if these *cis*-elements are involved in stress responses experimentally. In addition, the *cis*-element mapping in the promoters likely has high false positive and negative rates. Therefore, to increase the confidence in *cis*-element mapping, we focused on the 47 elements significantly enriched in the promoter sequences of stress responsive genes (Figure 6A).

Putative ancestral *cis*-element content of duplicate genes was estimated similar to the inference of ancestral stress responses (see Methods). We found that some *cis*-elements are enriched in genes that are responsive to nearly all conditions while the others have an extremely narrow response spectrum (Figure 6A). The presence of putative *cis*-elements that are involved in multiple stress responses indicates that elimination of any of these elements could lead to asymmetric partitioning of stress responses among duplicate pairs. In fact, we found that the duplicate pairs with asymmetric partitioning (*Asy* = 1) have significantly more “broadly responsive” *cis*-elements than the pairs that experienced symmetric partitioning (*Asy* = 0) (p<3.3E-08, Fisher's exact test). A putative *cis*-element is defined as broadly responsive if it belongs to the top 25^th^ percentile of the distribution of the number of conditions the elements are enriched in. Nonetheless, these are several potentially condition-specific *cis*-elements suggesting that asymmetry in stress response can be correlated with the asymmetric elimination of condition *cis*-elements in duplicate pairs.

Based on an earlier study that found a significant positive correlation between the density of *cis*-elements and the number of conditions in which a gene was differently regulated [68], we hypothesized that the daughter gene with more subfunctions would have more *cis*-elements compared to the other daughter gene with fewer or no ancestral subfunctions. Such correlation can be examined by comparing the subfunction and *cis*-element asymmetries. We first examined the pattern of partitioning of putative, stress responsive *cis*-elements among duplicates and found that partitioning tends to be extremely asymmetric (data not shown). Most importantly, there is a significant positive correlation between asymmetry of stress responsive *cis*-element content and asymmetry in stress responses among duplicate pairs (Figure 6B). This correlation is the most striking when *Asy* = 1 or −1 in both *cis*-element and stress response partitioning. Although *cis*-regulatory motifs may only explain ∼3% of expression divergence between duplicate genes [65] and our *cis*-element mapping is tentative, our findings indicate that the extremely asymmetric partitioning of stress responses between duplicates can be partly explained by the asymmetric elimination of *cis*-elements, especially those that are broadly responsive.

### Relationship between stress response partitioning symmetry and gain-of-function

**Figure 7:**
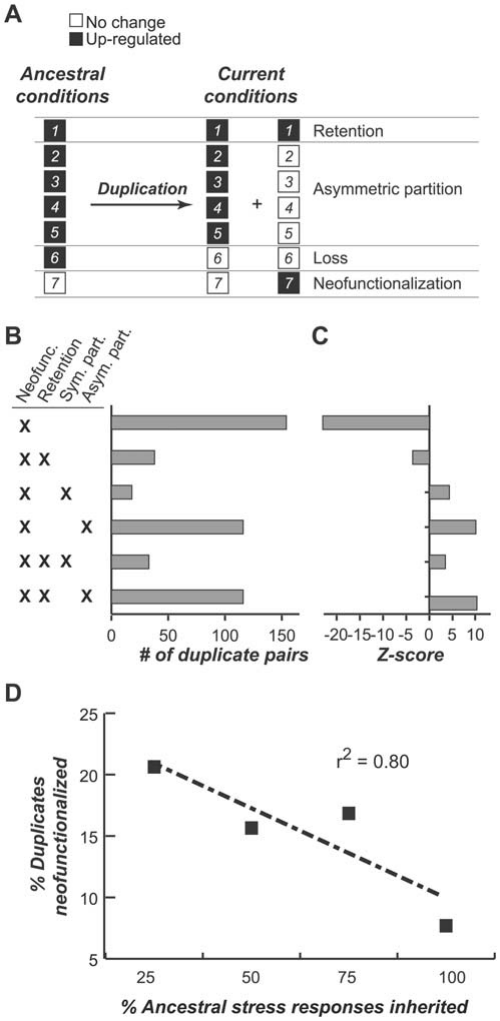
Co-occurrence of neofunctionalization and other scenarios of stress response evolution in duplicate pairs. (A) Example of how stress responses may be partitioned among duplicate pairs. Retention, symmetric partitioning (*Asy*<1), and asymmetric partitioning (*Asy* = 1) are as defined in Figure 5A. Note that only up-regulation is considered here. For down-regulated genes, see Figure S6. (B) Number of duplicate pairs observed for each combination of evolutionary scenarios involving neofunctionalization. (C) Degrees of deviation (Z-score) in the observed number of duplicate pairs for each combination compared to simulated data consisting of duplicate pairs with randomly assigned extant gene responses. The Z-score indicates how many standard deviations an observation is above or below the mean of the simulated distribution. (D) Relationship between the percentage of duplicated gene copies with ≥1 stress response gain (neofunctionalization) and the percentage of ancestral stress responses inherited. The dotted line represents the linear fit.

Duplicates with symmetric partitioning (defined as duplicate pairs with *Asy*<1, Figure 5A) of ancestral stress responses are clear examples of subfunctionalization and were likely retained because both copies complement each other. In contrast, neofunctionalization (gain of function) may play a more important role than subfunctionalization in retention of duplicates with extreme asymmetric stress response partitioning (*Asy* = 1, Figure 5A). If neofunctionalization is more important for the retention of asymmetrically partitioned duplicates than for symmetrically partitioned duplicates, we would expect to see a corresponding over-representation in the number of stress response gains among the asymmetrically partitioned duplicates. To test this, we examined the frequency of stress response gains in the context of several other evolutionary scenarios (Figure 7A) including parallel retention and parallel loss (Figure 4A) and partitioning (symmetric and asymmetric, Figure 5A).

Most duplicate pairs with stress response gains (neofunctionalization) either have no other informative subfunction or have subfunctions that were partitioned asymmetrically (Figure 7B). Relatively few neofunctionalized duplicate pairs have experienced symmetric partitioning or parallel retention. This pattern was expected because (1) there are substantially more duplicate pairs with some degree of subfunction partitioning than with parallel retention (Figure 4B) and (2) among duplicate pairs with subfunction partitioning, there are substantially more duplicate pairs with extreme asymmetry (Figure 5B). It is not clear if significantly more duplicates exhibit “pure” neofunctionalization (gain responsiveness for a stress but do not have any other ancestral stress responses) or if neofunctionalization tends to occur in duplicate genes with subfunction partitioning. To address these questions, we permuted the stress responses among the duplicate pairs while fixing the ancestral conditions to determine whether the observed numbers of duplicate pairs exhibiting each scenario are significantly over or under-represented (Figure 7C).

Interestingly, duplicates with stress response gains (or neofunctionalization) tend to be those that experienced either symmetric or asymmetric partitioning. Neofunctionalization among duplicates exhibiting symmetric partitioning is consistent with the “sub-neofunctionalization” model where subfunctionalization contributes to the initial retention of duplicates, which then gain advantageous mutations over time [21],[38]. However, duplicates with neofunctionalization and asymmetric partitioning have a higher overall Z-score (Z = ∼10, *p*<1E-6) compared to those with symmetric partitioning (Z = ∼4, *p*<5E-3). The significant co-occurrence of duplicate pairs with asymmetric partitioning and stress response gain highlights the intriguing possibility that gain-of-responsiveness has contributed to retention of the duplicate copies that did not inherit any subfunction. Consistent with this possibility, we also found that, among the 230 duplicate pairs experiencing both neofunctionalization and asymmetric partitioning, neofunctionalization occurred on the duplicate copy with no subfunctions in 71% of the cases (Fisher's Exact Test, *p*<4.2E-6). Furthermore, the percentage of duplicates that maintained ancestral stress response is negatively correlated with the percentage of duplicates that gained new functions (*r* ^2^ = 0.80, *p*<0.10 Figure 7D). A similar trend is also observed when considering down-regulated duplicates (Figure 6C), although the correlation is weaker (*r* ^2^ = 0.55, *p*<0.22). Although we do not have direct evidence demonstrating the fitness advantage in gaining stress responses, our finding that neofunctionalized duplicates tend to be those that inherited no ancestral stress response suggests that some of these gains have contributed to the retention of duplicates and are likely adaptive.

## Discussion

Mounting the proper responses to stressful environmental conditions is central to the survival of living organisms. Given the transient and variable nature of environmental stimuli and the fact that plants cannot escape stressful environments through movement, the strong selection pressure imposed by stress conditions likely leads to frequent turnover (gains and losses) of stress responses among plant genes. In this study, we explored the extent and patterns of stress response turnover among duplicate genes in *A. thaliana* based on their expression patterns under stress conditions. We presented evidence that duplicate genes experienced substantial changes in stress responses over time and have likely contributed to the physiological complexity in plants. Similarly, it has been argued that plant duplicate genes play pivotal roles in the morphological complexity [6].

Unlike earlier studies of regulatory evolution, we estimated ancestral expression patterns under stress conditions, which allowed us to differentiate between retention, loss, gain, and switch of stress responses. Although ancestral state reconstruction has been widely employed in evolutionary studies, few published studies have applied the reconstruction method for understanding evolution of gene function [41]. Currently there are two ways to evaluate the performance of reconstruction methods. The first is based on some evidence of ancestral states, such as fossil record [70]. Another approach, widely applied in ancestral sequence reconstruction, is to conduct simulation studies based on a pre-existing model, such as any substitution model in protein evolution [71]. Unfortunately, neither ancestral evidence nor a model of functional state evolution is available for us to validate the inferred ancestral states. We should point out that this is the major deficiency in our study.

Nonetheless, much of what we found here is independent of ancestor reconstruction and consistent with some fundamental theories in duplicated gene evolution. Comparing the stress responses of extant genes to their most recent ancestor, we found that the evolution of stress responses likely involves an initial accelerated rate of both loss and gain. This is potentially due to the combined action of neutral and/or positive selection followed by a period of strong purifying selection. This pattern is reminiscent of the selection intensity (*Ka/Ks* as proxy) profile of duplicate gene coding sequences over time (*Ks* as proxy) where younger duplicates have substantially more relaxed selection compared to older duplicates. In addition to timing of duplication, we found that duplication mechanisms influence stress response evolution; tandem duplicates in general are more likely to gain or lose stress responsiveness compared to non-tandem duplicates. We reason that this is because tandem duplication does not ensure duplication of the entire promoter and relevant *cis*-regulatory control mechanisms. Another important finding is that, when *Ks*>0.8, more genes retain (∼60%) ancestral stress responses than lose (∼40%) them, which indicates that some stress response functions may be retained in the gene for a long time.

When examining duplicate gene pairs, we found that in a substantial number of cases the stress responses are retained in both duplicates, even those duplicated hundreds of millions of years ago. Nonetheless, despite these interesting cases of parallel retention, partitioning of ancestral stress responses is the predominant fate. In particular, ancestral response partitioning occurred in a highly asymmetric fashion between duplicates where one duplicate apparently inherited no subfunction (stress response). How can duplicates that inherit no ancestral subfunction be retained? Based on two lines of evidence, we show that their retention may be due to gain of new functions. First, we found that duplicate pairs with asymmetric response partitioning tend to have a significantly higher number of stress response gains compared to those with symmetric partitioning. Secondly, duplicates without any inherited stress response subfunctions are over-represented among duplicates with stress response gains. Our findings are consistent with the sub-neofunctionalization hypothesis [21] and provide additional evidence that neofunctionalization tends to take place in duplicate copies with few or no inherited subfunctions. Finally, we found that asymmetry in ancestral stress response partitioning is correlated with the partitioning of predicted stress responsive *cis*-elements, indicating that differences in *cis*-element content between duplicates may contribute to stress response asymmetry. We should point out that, in addition to *cis*-regulatory element content, there are multiple other sources of variation that may impact gene expression evolution. For example, genome doubling and hybridization usually lead to rapid and drastic changes in gene expression (for review, see [72]). Epigenetic state has also most likely had a significant influence on the functional divergence of duplicate genes [6]. Further studies will be required to examine the effects of epigenetic phenomena on expression divergence.

Based on of the relationship between stress response partitioning patterns and neofunctionalization, we anticipate that neofunctionalization in the form of stress response gains may contribute to the retention of stress responsive duplicate genes and that some of these functional gains are adaptive. Nonetheless, there are at least two major issues that require further, detailed investigation. The first is the relationship between gain and loss of stress responses and gain and loss of putative stress responsive *cis*-elements. Given that the collection of *cis*-elements involved in stress response remains incomplete and *cis*-element mapping is typically associated with high false positive and negative rates, it will be necessary to make use of the abundant microarray data to uncover sequence motifs and establish their roles in modulating stress response at the level of gene expression. Secondly, it remains to be demonstrated that these changes in gene expression and in *cis*-regulatory regions indeed have measurable effects on the fitness of plants under stress conditions. Future studies in these two areas will provide new insight into the mechanisms leading to differences in stress response between genes and into how these differences contributed to the survival of organisms under stress.

## Methods

### Stress expression data

Stress expression microarray data were obtained from AtGenExpress (http://www.uni-tuebingen.de/plantphys/AFGN/atgenex.htm) and included 8 abiotic (cold, drought, genotoxic, heat, osmotic, salt, UV-B, wounding) and 8 biotic (DC3000, Flg22, GST-NPP1, HrcC, HrpZ, P.infestans, Psph, avrRpm1) stress conditions with treatment time points ranging from 0.5 to 24 hours. The array intensities were background corrected and quantile normalized with functions in the affy package of Bioconductor (www.bioconductor.org [73]). LIMMA was used to compare hybridization intensities of treated samples against their corresponding controls [74]. Up- and down-regulated genes under each stress condition/time were defined as those with significantly higher and lower hybridization intensities, respectively, (at 5% false discovery rate) for stress treatments than control treatments. Non-responsive genes were defined as those without a significant change in expression upon stress treatment.

### Gene family definition, sequence alignments, and phylogeny inference

To define gene families in *A. thaliana*, an all against all similarity search of *A. thaliana* annotated protein sequences (TAIR, v7) was conducted using BLAST [75] with an E-value cutoff of 1e-5. Based on transformed E-values, we generated similarity clusters representing gene families with the Markov Clustering program (http://micans.org/mcl/). Multiple protein sequence alignments for each gene family were generated with ClustalW ([76], Blosum 62 matrix, 5.0 gap opening and 10.0 gap extension penalty). Based on the alignment, protein distances among genes in a family were estimated with the PRODIST program in the PHYLIP package (JTT substitution matrix and gamma correction with a coefficient of variation of 0.3126, [77]). This protein distance matrix was then used to generate a Neighbor-joining tree which was rooted at the mid-point with PHYLIP. Trees with >50 taxa were subdivided into sub-trees where (1) the base nodes of the sub-trees were 0.05 distance units away from the based node of the family tree and (2) each sub-tree contains ≤50 taxa. The sub-trees were subdivided repeatedly until both criteria were met.

For each “family” (cluster with ≤50 members) or “subfamily” (represented by a qualified sub-tree), a consensus tree was inferred using MrBayes [42] with the protein mixed model and a Neighbor-Joining guide tree. First we ran two chains for 1×10^7^ iterations, sampled every 10 iterations and halted when average standard deviation of split frequencies was <0.01. For families/subfamilies that did not converge at this point, the program was re-run with 4 chains (Markov Chain Monte Carlo sampling) for 1×10^6^ iterations, sampled every 1,000 iterations, halted when the average standard deviation of split frequencies was <0.01. If at the end of the run the average standard deviation was <0.05 and stable, we considered the sampling of the posterior distribution to be adequate. Trees generated after the “burn in” point (the first 25% of the resulting trees were discarded) were used to build a consensus tree using the sumt function in MrBayes by including all compatible groups.

### Reconstruction of ancestral stress response states

Reconstruction of ancestral stress response states was performed with BayesTraits ([45]) where the transition rates were estimated with maximum likelihood (ML), assuming that the probability of response change is proportional to the branch length. Since the rate of forward transition (no response→up or down regulation) is likely different from the rate of reverse transition (up or down regulation→no response) [41], the asymmetrical 2-parameters model in BayesTraits was used. For each up- and down-regulated gene identified, we defined three discrete functional states for each condition/time: up-regulation (1), down-regulation (−1) and no change (0). The BayesTraits run was done with the method “Most Recent Common Ancestor” for each condition/time and each consensus tree. To assess the significance of the ancestral function prediction, we only used ancestral states with posterior probability >0.5. There were a few cases where all the genes in one tree had the same functional states and BayesTraits could not be used. In these cases, we assumed that all the internal nodes in these trees had the same functions.

### Analysis of WGD and tandem duplicates

Genes derived by WGD were defined based on an earlier study [8]. Tandem duplicated genes were defined as genes in any gene pair, T_1_ and T_2_ that (1) belong to the same domain family, (2) are located within 100 kb each other and (3) are separated by ≤10 non-homologous spacer genes. If a gene G qualifies as both WGD and tandem duplicates, G is classified as tandem only if the most recent duplication event involving G (based on the gene family phylogeny) is tandem.

### Analysis of *cis*-elements

The *cis*-elements and their putative locations in *A. thaliana* promoters were obtained from AGRIS [78]. In this study, we only used predicted *cis*-elements located within 1000 bp upstream of the transcriptional start site (putative promoter). In addition, *cis*-elements were included only if they were over-represented in the putative promoter regions of genes that were responsive to ≥1 conditions. Over-representation of *cis*-elements among responsive genes was determined by setting up a 2-by-2 contingency table for each *cis*-element-condition/time combination and testing for significance using the chi-square test.

## Supporting Information

Figure S1 Influence of ML model parameters on ancestral state reconstruction. Likelihood Ratio (LR) is defined as the absolute value of 2[log(L(model1))−log(L(model2))] (L∶Likelihood). Here we compared models with κ = 0 vs. free κ and κ = 5 vs. free κ (BayesTraits [45]). Here the parameter κ>1 will stretch the longer branches more than the shorter branches. At κ = 0, no assumption was made about the correlation between sequence and character state evolution. LR is distributed as a χ^2^ distribution with one degree of freedom. We found that 95% of the ancestral states reconstructed are not significantly different.
(0.24 MB PDF)

Figure S2 Relationship between stress evolution scenarios and Ks for abiotic and biotic conditions. The relative frequencies of the different stress response evolution scenarios (as shown in Figure 1) vary as Ks increases. (A) The relative frequency of external branches with retention, gain, and switch of down-regulation under abiotic stress conditions. (B) The relative frequency of external branches with retention, loss, and switch of down-regulation under abiotic stress conditions. (C) The relative frequency of external branches with retention, gain, and switch of up-regulation under biotic stress conditions. (D) The relative frequency of external branches with retention, loss, and switch of up-regulation under biotic stress conditions. (E) The relative frequency of external branches with retention, gain, and switch of down-regulation under biotic stress conditions. (F) The relative frequency of external branches with retention, loss, and switch of down-regulation under biotic stress conditions. (G) The relative frequencies of external branches with retention, gain, and switch (a), and retention, loss, and switch (b) are plotted for individual conditions (Ga-AHb). Black line: retention, red: loss, green: gain, switch: blue.
(2.72 MB PDF)

Figure S3 Extent of stress response loss differs between duplicates arising from different duplication mechanisms (down-regulation). Comparison of the extent of loss of down-regulation under abiotic (A) and biotic (B) stress conditions for genes derived from tandem and non-tandem mechanisms. Asterisks indicate significant differences based on Wilcoxon rank sum tests (p<0.05).
(0.29 MB PDF)

Figure S4 Stress response evolution in duplicate pairs (down-regulation). (A) The observed frequency of each scenario shown in Figure 4A when ancestral states were considered. The bar plots indicate the observed frequency and the box plots indicate the frequency distributions of random scenarios. The random scenarios were generated by assigning the extant genes to stress responses randomly and determining the frequency of each stress response scenario over 10,000 runs. (B) The observed frequency of each scenario shown in Figure 4A without considering ancestral states. The box plots were generated based on the same randomization scheme as in (A).
(0.26 MB PDF)

Figure S5 Extreme asymmetry in ancestral stress response partitioning among duplicates (down-regulation). (A) Relative frequencies of the number of duplicate pairs with various degrees of asymmetric partitioning with ancestor reconstruction. (B) Over-representation of duplicate pairs with extremely asymmetric partitioning of stress responses with ancestral states taken into consideration. Log ratio (base 2) between the observed and expected number of duplicate pairs for each number of condition combinations was used as a measure of over- (red) and under-representation (blue) in the contour plot. The expected numbers were generated based on random binomial sampling. (C) Relative frequencies of the number of duplicate pairs with various degrees of asymmetric partitioning without ancestor reconstruction. (D) Contour plot of over-representation of duplicate pairs with extremely asymmetric partitioning of stress responses without ancestor reconstruction.
(0.59 MB PDF)

Figure S6 Co-occurrence of gain (neofunctionalization) and other scenarios of stress response evolution in duplicate pairs (down-regulation). (A) Number of duplicate pairs with different combinations of stress response evolution scenarios when considering down-regulation. Retention, symmetric partitioning (*Asy<1*), and asymmetric partitioning (*Asy = 1*) are as defined in Figure 5A. (B) Degrees of deviation (Z-score) of observed number of duplicate pairs exhibiting a stress response evolution scenario combination compared to simulated data consisting of duplicate pairs with randomly assigned extant gene responses. (C) Relationship between the percentage of duplicated genes with ≥1 stress response gain (neofunctionalization) and the percentage of ancestral stress responses inherited. The dotted line represents the linear fit.
(0.33 MB PDF)

Table S1 Frequency of stress response evolution scenarios of ancestral-extant gene pairs. Numbers of external branches exhibiting four possible evolutionary scenarios under each condition/time.
(0.96 MB PDF)
