# Supplementary figures and images for: Evolution of Stress-Regulated Gene Expression in Duplicate Genes of Arabidopsis thaliana
Source: PLoS Genet. 2009 Jul 31;5(7):e1000581. doi: 10.1371/journal.pgen.1000581 (PMC2709438; doi:10.1371/journal.pgen.1000581)

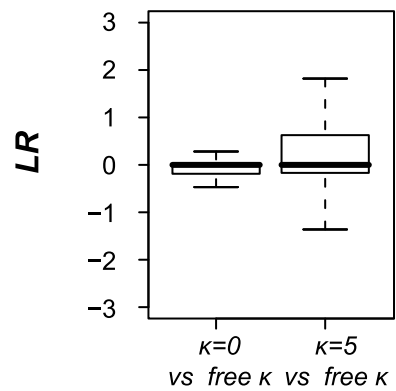

Supplement: Figure S1 — Influence of ML model parameters on ancestral state reconstruction. Likelihood Ratio (LR) is defined as the absolute value of 2[log(L(model1))−log(L(model2))] (L∶Likelihood). Here we compared models with κ = 0 vs. free κ and κ = 5 vs. free κ (BayesTraits [45]). Here the parameter κ>1 will stretch the longer branches more than the shorter branches. At κ = 0, no assumption was made about the correlation between sequence and character state evolution. LR is distributed as a χ2 distribution with one degree of freedom. We found that 95% of the ancestral states reconstructed are not significantly different. (0.24 MB PDF) [file pgen.1000581.s001.pdf]

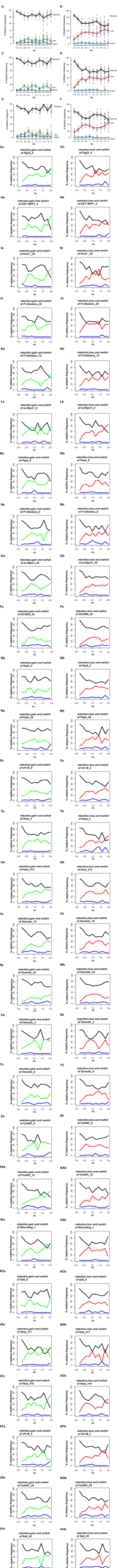

Supplement: Figure S2 — Relationship between stress evolution scenarios and Ks for abiotic and biotic conditions. The relative frequencies of the different stress response evolution scenarios (as shown in Figure 1) vary as Ks increases. (A) The relative frequency of external branches with retention, gain, and switch of down-regulation under abiotic stress conditions. (B) The relative frequency of external branches with retention, loss, and switch of down-regulation under abiotic stress conditions. (C) The relative frequency of external branches with retention, gain, and switch of up-regulation under biotic stress conditions. (D) The relative frequency of external branches with retention, loss, and switch of up-regulation under biotic stress conditions. (E) The relative frequency of external branches with retention, gain, and switch of down-regulation under biotic stress conditions. (F) The relative frequency of external branches with retention, loss, and switch of down-regulation under biotic stress conditions. (G) The relative frequencies of external branches with retention, gain, and switch (a), and retention, loss, and switch (b) are plotted for individual conditions (Ga-AHb). Black line: retention, red: loss, green: gain, switch: blue. (2.72 MB PDF) [file pgen.1000581.s002.pdf]

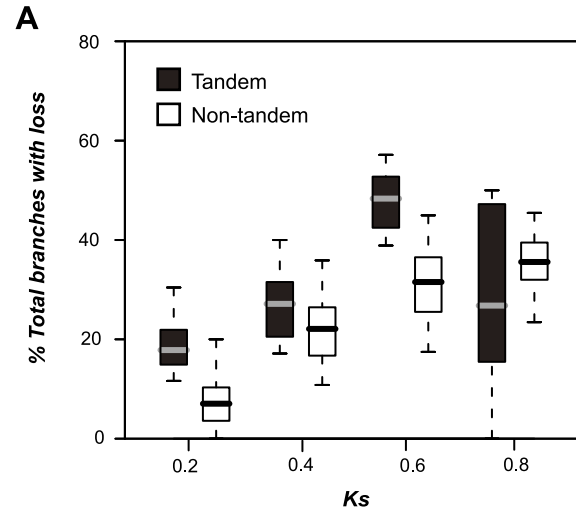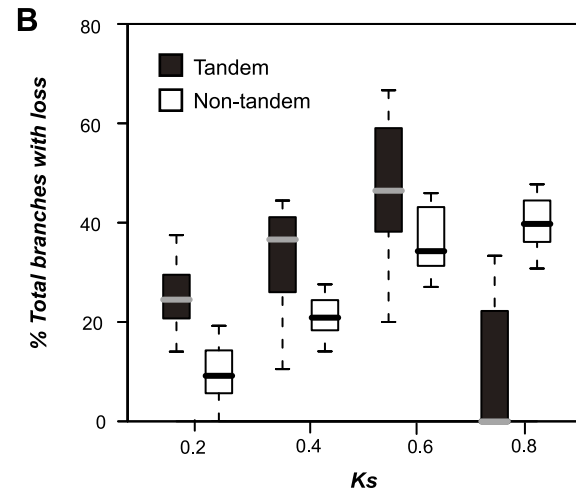

Supplement: Figure S3 — Extent of stress response loss differs between duplicates arising from different duplication mechanisms (down-regulation). Comparison of the extent of loss of down-regulation under abiotic (A) and biotic (B) stress conditions for genes derived from tandem and non-tandem mechanisms. Asterisks indicate significant differences based on Wilcoxon rank sum tests (p<0.05). (0.29 MB PDF) [file pgen.1000581.s003.pdf]

**A**

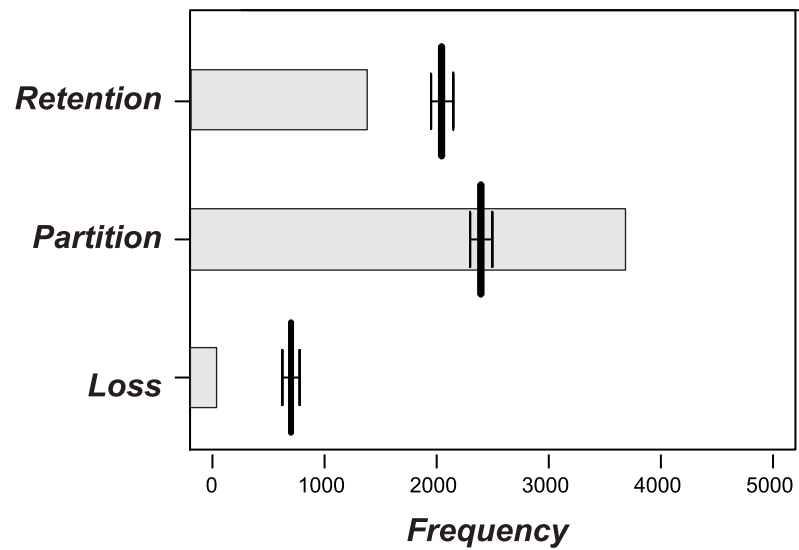

**B**

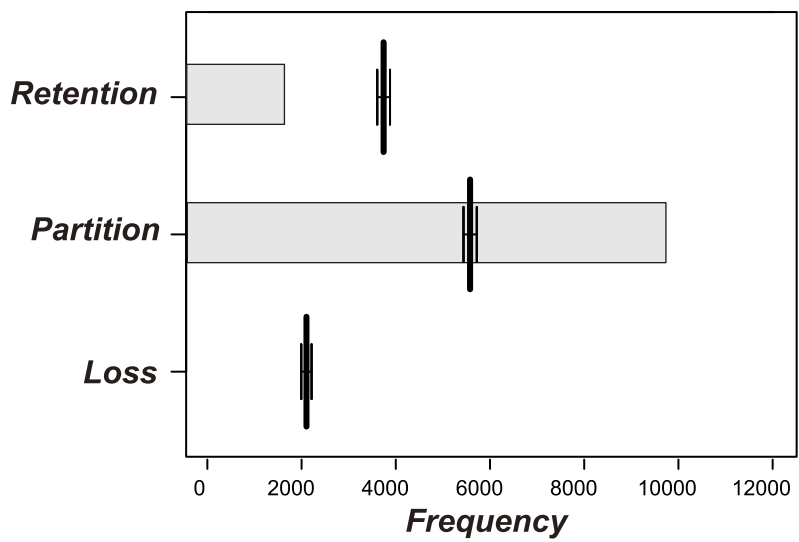

Supplement: Figure S4 — Stress response evolution in duplicate pairs (down-regulation). (A) The observed frequency of each scenario shown in Figure 4A when ancestral states were considered. The bar plots indicate the observed frequency and the box plots indicate the frequency distributions of random scenarios. The random scenarios were generated by assigning the extant genes to stress responses randomly and determining the frequency of each stress response scenario over 10,000 runs. (B) The observed frequency of each scenario shown in Figure 4A without considering ancestral states. The box plots were generated based on the same randomization scheme as in (A). (0.26 MB PDF) [file pgen.1000581.s004.pdf]

**A**

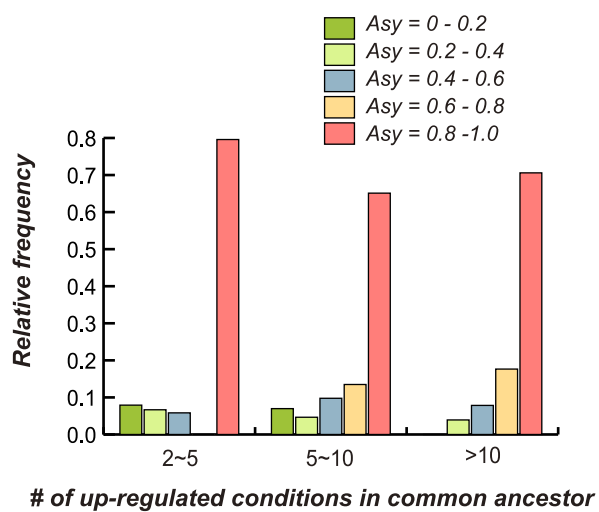

**B**

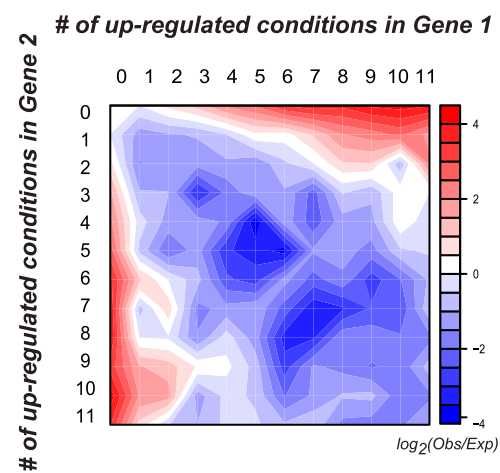

**C**

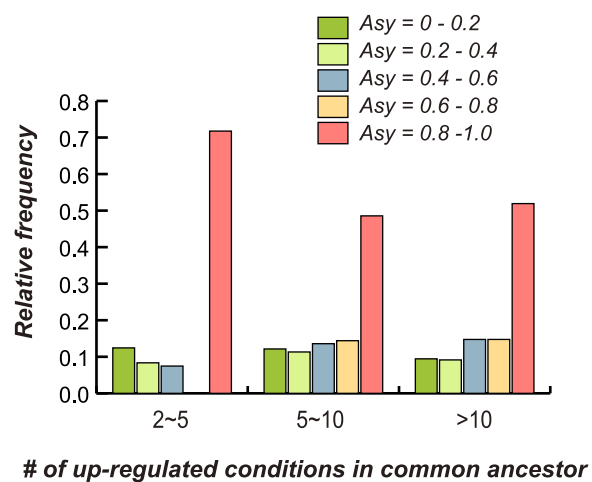

**D**

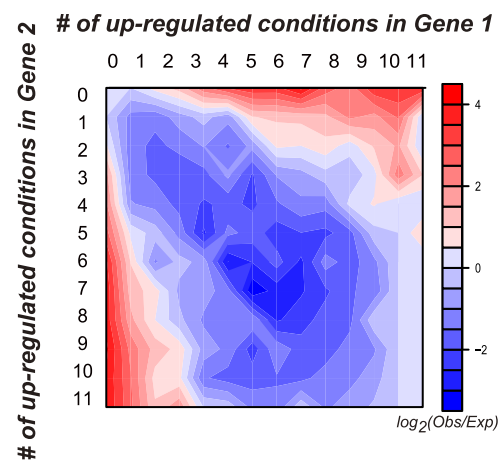

Supplement: Figure S5 — Extreme asymmetry in ancestral stress response partitioning among duplicates (down-regulation). (A) Relative frequencies of the number of duplicate pairs with various degrees of asymmetric partitioning with ancestor reconstruction. (B) Over-representation of duplicate pairs with extremely asymmetric partitioning of stress responses with ancestral states taken into consideration. Log ratio (base 2) between the observed and expected number of duplicate pairs for each number of condition combinations was used as a measure of over- (red) and under-representation (blue) in the contour plot. The expected numbers were generated based on random binomial sampling. (C) Relative frequencies of the number of duplicate pairs with various degrees of asymmetric partitioning without ancestor reconstruction. (D) Contour plot of over-representation of duplicate pairs with extremely asymmetric partitioning of stress responses without ancestor reconstruction. (0.59 MB PDF) [file pgen.1000581.s005.pdf]

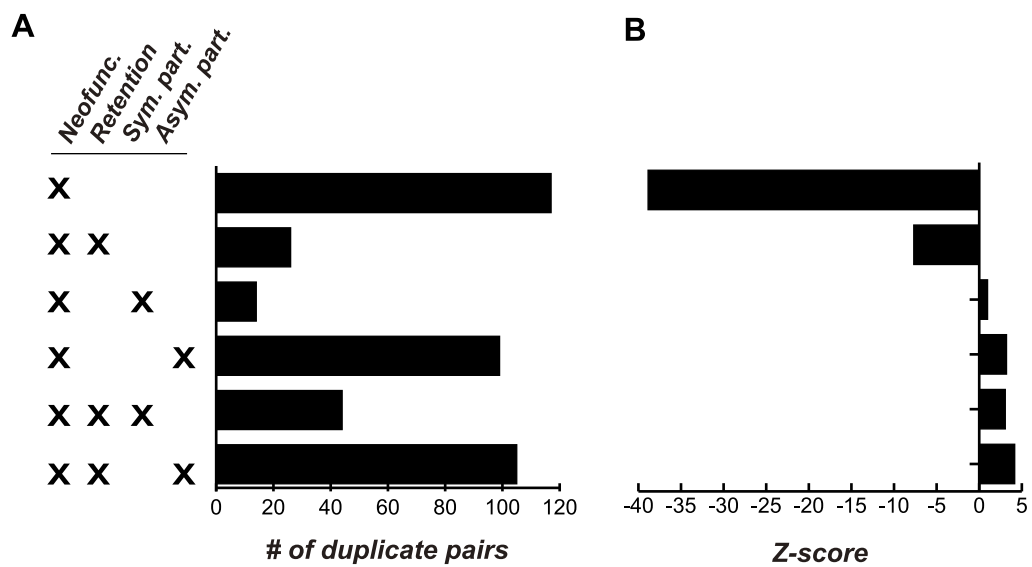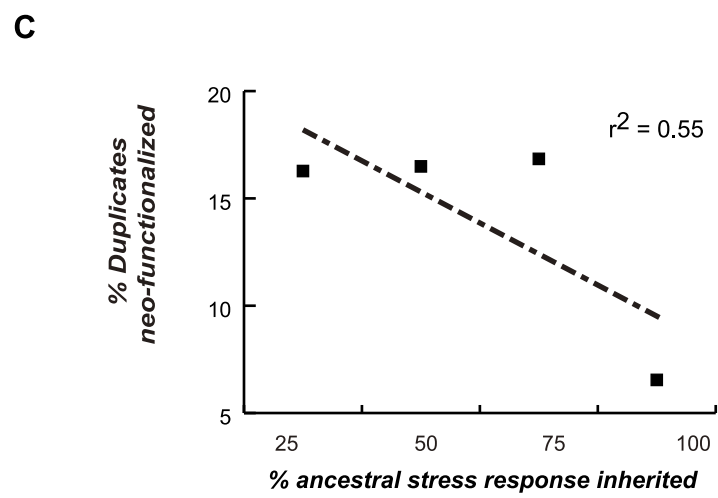

Supplement: Figure S6 — Co-occurrence of gain (neofunctionalization) and other scenarios of stress response evolution in duplicate pairs (down-regulation). (A) Number of duplicate pairs with different combinations of stress response evolution scenarios when considering down-regulation. Retention, symmetric partitioning (Asy<1), and asymmetric partitioning (Asy = 1) are as defined in Figure 5A. (B) Degrees of deviation (Z-score) of observed number of duplicate pairs exhibiting a stress response evolution scenario combination compared to simulated data consisting of duplicate pairs with randomly assigned extant gene responses. (C) Relationship between the percentage of duplicated genes with ≥1 stress response gain (neofunctionalization) and the percentage of ancestral stress responses inherited. The dotted line represents the linear fit. (0.33 MB PDF) [file pgen.1000581.s006.pdf]
