# Supplementary material for: Evolution of Stress-Regulated Gene Expression in Duplicate Genes of Arabidopsis thaliana
Source: PLoS Genet. 2009 Jul 31;5(7):e1000581. doi: 10.1371/journal.pgen.1000581 (PMC2709438; doi:10.1371/journal.pgen.1000581)
Supplement: Table S1 — Frequency of stress response evolution scenarios of ancestral-extant gene pairs. Numbers of external branches exhibiting four possible evolutionary scenarios under each condition/time. (0.96 MB PDF) [file pgen.1000581.s007.pdf]

	1 → 1	-1 → -1	1 → 0	-1 → 0	0 →1	0 →-1	1 → -1	-1 →1
avrRpm1_2	26	4	25	3	2	0	0	0
avrRpm1_24	541	336	184	131	59	24	18	11
avrRpm1_6	279	136	132	90	31	17	4	4
Cold4C_12	440	410	153	158	49	61	21	27
Cold4C_24	506	540	165	187	59	59	37	20
Cold4C_3	194	41	113	24	12	6	1	0
Cold4C_6	358	151	178	79	32	24	10	8
DC3000_2	124	44	92	28	5	3	0	0
DC3000_24	646	539	186	168	68	49	33	38
DC3000_6	53	25	38	20	7	3	2	0
Drought_3	49	4	39	3	3	0	0	1
Drought_6	108	30	61	27	4	1	0	0
Flg22_1	196	19	116	13	22	6	0	0
Flg22_4	283	167	132	87	24	23	7	2
Genotoxic_12	22	7	18	7	4	2	0	0
Genotoxic_6	24	8	19	10	1	0	0	0
GST-NPP1_1	67	3	46	3	12	0	0	0
GST-NPP1_4	256	120	119	67	20	16	3	1
Heat_0.25	32	196	18	111	7	22	1	1
Heat_0.5	3	30	5	21	1	5	0	0
Heat_1	151	236	62	140	7	31	1	8
Heat_3	224	471	93	217	22	60	12	7
Heat_3+1	271	419	107	220	26	43	8	5
Heat_3+21	32	119	27	68	2	8	3	0
Heat_3+3	232	336	100	153	33	38	9	9
Heat_3+9	188	222	96	119	30	16	2	5
HrcC-_2	68	11	36	7	5	0	0	0
HrcC-_24	346	179	136	84	35	21	10	6
HrcC-_6	81	47	38	35	4	3	0	2
HrpZ_1	196	13	112	9	10	4	1	0
HrpZ_4	464	296	192	123	48	30	14	11
Osmotic_1	45	6	32	5	5	1	0	0
Osmotic_12	501	531	191	161	48	50	13	37
Osmotic_24	665	665	206	152	63	68	24	36
Osmotic_3	406	197	175	89	50	28	5	3
Osmotic_6	479	399	175	140	57	43	13	26
P.infestans_12	285	188	138	88	19	29	6	6
P.infestans_24	261	223	148	120	24	22	2	4
P.infestans_6	363	367	162	163	24	44	16	12
Psph_2	83	5	55	2	14	2	0	0
Psph_24	422	297	152	111	49	30	18	12
Psph_6	312	203	119	107	22	24	4	4
Salt_12	80	45	42	27	5	8	3	1
Salt_24	390	297	155	137	33	31	19	7
Salt_3	163	93	82	45	15	12	2	3
Salt_6	292	140	131	70	23	13	7	3
UV-B_0.25	38	36	27	28	2	3	0	0
UV-B_1	266	87	124	55	25	10	2	4
UV-B_12	211	94	100	56	22	9	4	3
UV-B_24	224	96	102	47	26	13	1	1
UV-B_3	469	443	172	173	40	34	7	19
UV-B_6	548	508	194	182	57	41	17	15
Wounding_0.25	29	7	19	3	3	1	0	0
Wounding_0.5	189	43	103	24	20	7	0	0
Wounding_1	294	104	144	53	25	15	1	1
Wounding_12	89	21	64	13	11	0	0	0
Wounding_3	66	4	42	2	5	0	0	0
